# Variation and assembly mechanisms of *Rhinolophus ferrumequinum* skin and cave environmental fungal communities during hibernation periods

**DOI:** 10.1128/spectrum.02233-24

**Published:** 2025-01-23

**Authors:** Haixia Leng, Aoqiang Li, Zhongle Li, Joseph R. Hoyt, Wentao Dai, Yanhong Xiao, Jiang Feng, Keping Sun

**Affiliations:** 1 Jilin Provincial Key Laboratory of Animal Resource Conservation and Utilization, Northeast Normal University, Changchun, China; 2 Key Laboratory of Vegetation Ecology, Ministry of Education, Changchun, China; 3 School of Life Sciences, Central China Normal University, Wuhan, China; 4 College of Life Science, Jilin Agricultural University, Changchun, China; 5 Department of Biological Sciences, Virginia Polytechnic Institute, Blacksburg, Virginia, USA; China Agricultural University, Beijing, China

**Keywords:** *Pseudogymnoascus destructans*, fungal community, hibernation, environmental reservoir, dynamic change

## Abstract

Animal skin acts as the barrier against invasion by pathogens and microbial colonizers. Environmental microbiota plays a significant role in shaping these microbial communities, which, in turn, have profound implications for host health. Previous research has focused on characterizing microorganisms on bats’ skin and in their roosting environments, particularly bacterial communities. The emergence of white-nose syndrome, caused by the fungal-pathogen *Pseudogymnoascus destructans*, highlights the importance of understanding fungal dynamics in cave ecosystems and on bats’ skin. In this study, we employed ITS amplicon sequencing to investigate the fungal community associated with the skin of *Rhinolophus ferrumequinum* and surfaces within hibernacula. In addition, we utilized neutral community and null models to assess the relative importance of stochastic and deterministic processes in fungal community assembly. The infection status of *P. destructans* did not significantly impact fungal community composition either on bat skin or cave environments. However, fungal diversity was significantly higher in cave environments compared to bat skin. Notably, potentially inhibitory genera of fungal pathogens were present in both bats and cave environments during hibernation. Furthermore, the composition and structure of fungal communities on both bat skin and cave environments varied across hibernation periods. Our findings suggest neutral processes primarily drive the assembly of fungal communities associated with hibernating *R. ferrumequinum* and cave environments, with dispersal limitation exerting a significant influence. This study provides insights into the fungal communities associated with hibernating *R. ferrumequinum* and cave environments.

**IMPORTANCE:**

Animal habitats provide sources and reservoirs for host microorganisms, making it critical to understand changes in microbial communities between habitats and hosts. While most studies have focused on bacterial microorganisms, research on fungal communities is lacking. This study investigated how community dynamics and assembly processes differ between the skin of hibernating *Rhinolophus ferrumequinum* and the cave environments under pathogen stress. We found significant differences in the composition and structure of the fungal communities between bat skin and roosting cave environments. Fungal genera with potential inhibitory effects on pathogens were found in all bat skin and cave environments. In addition, dispersal limitations during stochastic processes were a key factor in the formation of environmental fungal communities on bat skin and in caves. These findings offer new insights for exploring pathogen-host-environment-microbe interactions.

## INTRODUCTION

White-nose syndrome (WNS) is an emerging infectious fungal disease caused by the fungus *Pseudogymnoascus destructans*, which primarily infects hibernating bats (1–4). This psychro-tolerant fungus colonizes bat wing membranes, leading to lesions and physiological disruption (2). Recent research indicates that *P. destructans* is widespread in North America (5–9), it has been documented in 40 states of the USA and 10 Canadian provinces (source: https://www.whitenosesyndrome.org/). A previous study also detected *P. destructans* in bats from China but with lower fungal loads compared to North America (10). Phylogenetic analysis suggests that *P. destructans* has coexisted with bats in Asia and Europe for a longer period, where bats may show greater resistance to the infection (11).

The skin acts as a critical physical barrier against pathogens and also plays a role in immune function while hosting a diverse microbiota (12). Research has demonstrated that exposure to *P. destructans* can modify the composition of bat skin microbiota, shifting in several bacterial taxa with antifungal properties (13–16). The skin is crucial for maintaining an animal’s health, and the surrounding environment, rich in various microorganisms, can significantly affect the host’s well-being. Therefore, understanding the impact of these microorganisms on the host’s health is essential (17). For instance, cave environments host various genera of antifungal bacteria, suggesting that these environments may serve as a source for such bacteria, which could colonize bat skin and provide protective effects (18).

Current research has largely focused on the effects of host bacteria on pathogens (14, 16, 17, 19), with limited studies on fungal-related symbiosis and environmental interactions. Bat caves, where bats hibernate, rest, and raise young, are critical for understanding fungal dynamics (20). The cave substrate harbors *P. destructans*, the fungal pathogen responsible for WNS (21). Fungi can grow on cave substrates, with their secondary metabolites providing nutrients for other fungi on rocky surfaces (22, 23). *Pseudogymnoascus destructans*, which persists in cave environments for long periods, is primarily spread by bats through pathogen shedding (24). Therefore, investigating fungal diversity and dynamic changes on the skin of hibernating bats and within bat hibernacula is vital for understanding their role in pathogen defense.

Recent theoretical advancements have highlighted the collaborative regulation of microbial community assembly by deterministic and stochastic processes, including drift and stochastic dispersal (25, 26). Understanding these mechanisms is crucial for comprehending fungal community dynamics. Neutral theory suggests that community structure results from stochastic processes, such as birth, death, colonization, extinction, and speciation, rather than species characteristics, assuming ecological adaptations as “neutral” (27). The Neutral Community Model (NCM) is a valuable tool for understanding these stochastic processes (28, 29). However, it does not specify the exact biological processes affecting community assembly. Thus, quantitatively identifying and defining these processes are essential. Stegen et al. proposed using a null model to assess the relative significance of different community assembly processes (30, 31). While neutral community and null models have been widely applied across various ecosystems, such as zebrafish (32), soil (33–35), sediments (36), rice (37), stoneflies (38), and human lung and human skins (39, 40). The forces shaping fungal communities on bat membrane skin remain poorly understood. Specifically, it is challenging to determine whether fungal community assembly on bat skin is influenced by selection for specific taxa in response to *P. destructans* infection pressure, which might confer resistance to pathogens.

The greater horseshoe bat (*Rhinolophus ferrumequinum*) is a common species in China, known for its notable resistance to *P. destructans* (10, 19). *Rhinolophus ferrumequinum* predominantly inhabits karst caves and large cave-like spaces year-round, exhibiting strong roost fidelity (41). During hibernation, from October to April (42), they wrap themselves in their wing membranes and hang upside down from the cave roof, often sleeping in clusters rather than singly (43). *Rhinolophus ferrumequinum* wakes irregularly during this period (44).

In this study, skin swabs from *R. ferrumequinum* and swabs from their hibernacula were collected to analyze the dynamics of fungal communities on bat skin during hibernation and to determine the processes driving the assembly of fungal communities on bat skin and in the environments. The objectives were to (i) investigate fungal infections and the dynamics of fungal communities on bat skin and within hibernacula environments during hibernation and (ii) reveal the importance of *P. destructans* infection as well as deterministic and stochastic processes shaping fungal communities on bat skin and in cave environments. This investigation sheds light on the dynamic changes observed on bat skin and in roosting cave environments during hibernation, thus enhancing our understanding of the complex interactions among hosts, environments, and pathogens in these ecosystems.

## RESULTS

### Fungal infections in hibernating bats and their hibernacula

**Fig 1:**
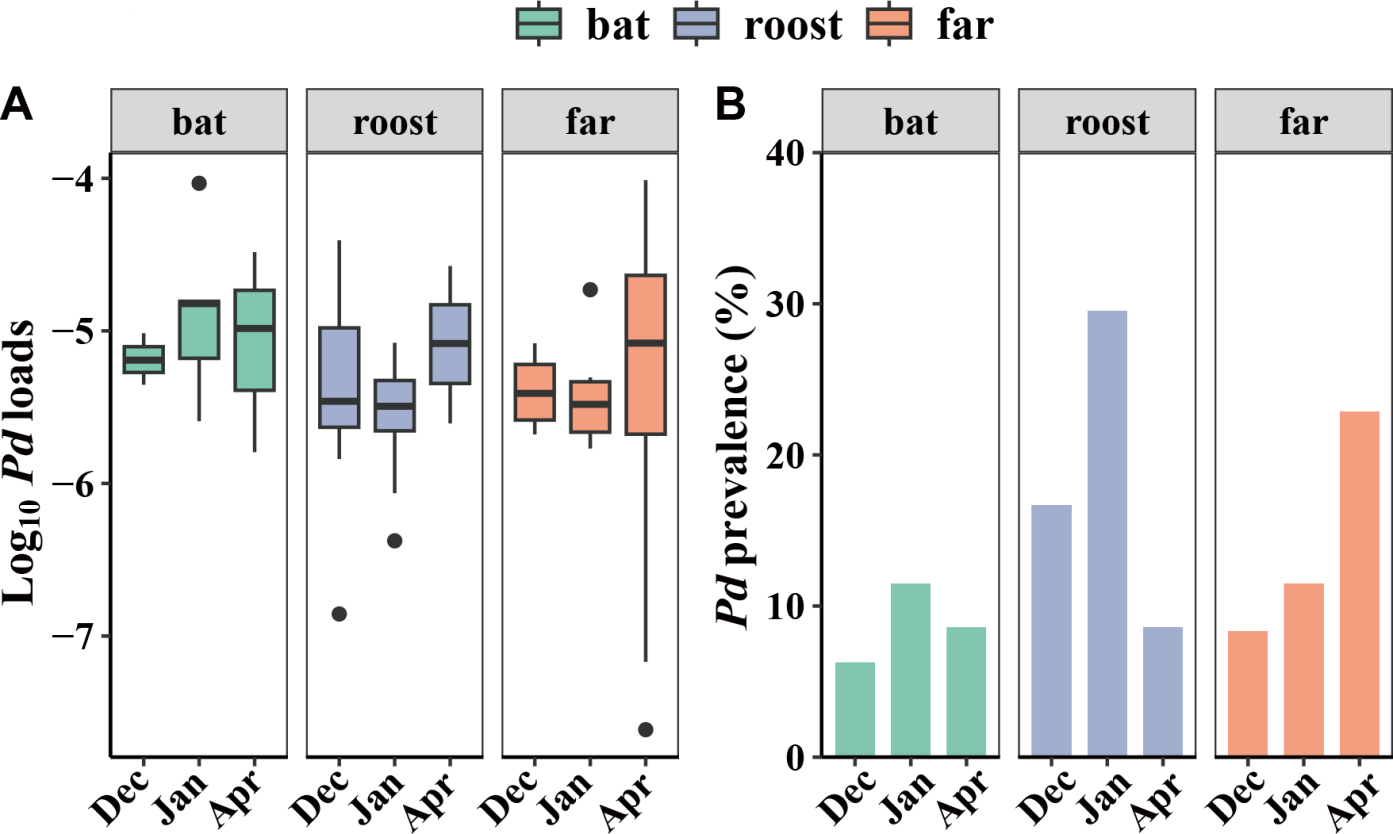
Prevalence and fungal loads of *Pseudogymnoascus destructans* (*Pd*) of infected individuals during a fungal infection in bats and cave environments at three time points during hibernation, December 2017 (Dec), January 2018 (Jan), and April 2018 (Apr): (**A**) *Pd* loads (log_10_) and (**B**) *Pd* prevalence. Figure 1A used a logarithmic modification on the *P. destructans* load value.

During hibernation, the average fungal load was highest in bat skin samples, followed by roost samples and then far samples, with no significant variation (Fig. 1A; Table S1). Overall, the *P. destructans* loads were relatively low during hibernation, averaging 4.15 e-06 ±1.12 e-05 (mean ± standard deviation) ng. No significant difference was observed among bat, roost, and far samples (Kruskal−Wallis test: χ^2^ = 10.785, *P* = 0.214) (Fig. 1A). At each hibernation stage, the prevalence of *P. destructans* was also relatively low across bat, roost, and far samples (Fig. 1B). However, the roost samples exhibited a higher fungal load compared to bat and far samples (Fig. 1A).

### Taxonomic composition of fungal communities from hibernating bats and their hibernacula

**Fig 2:**
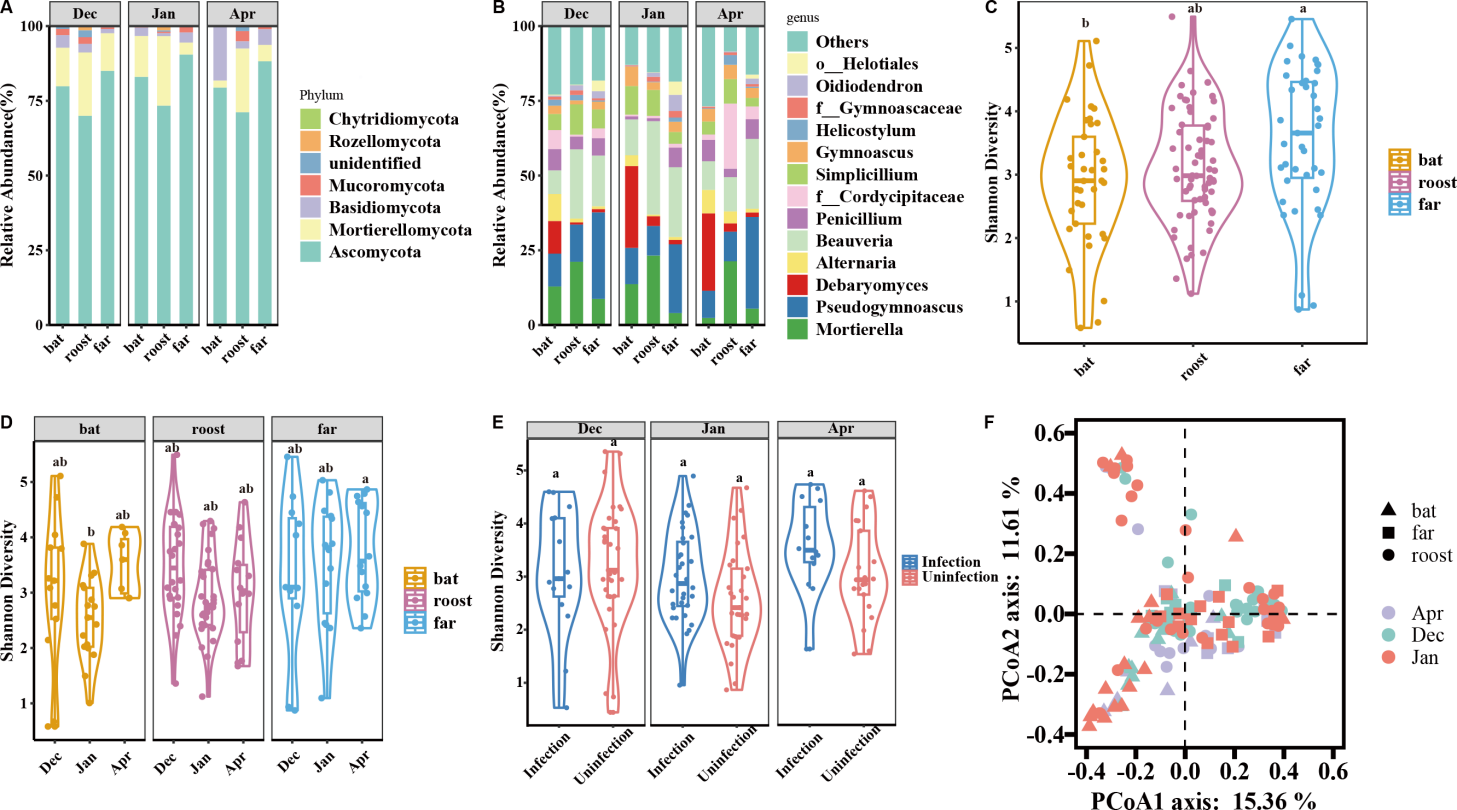
Fungal community composition and structure of skin surface of hibernating bats and their environments: species composition and average relative abundance at the (**A**) phylum and (**B**) genus levels (relative abundance >0.5%), (**C**) Shannon diversity of different samples; (**D**) Shannon diversity of fungal communities on the skin surface of hibernating bats and in the environments; (**E**) Shannon diversity of fungal communities of different infection status; and (**F**) Graph of principal coordinate analysis (PCoA) of Bray-Curtis dissimilarity for PCoA1 (x-axis) and PCoA2 (y-axis).

At the phylum level, fungal communities from hibernating bat, roost, and far samples were predominantly composed of Ascomycota, Mortierellomycota, Basidiomycota, and Mucoromycota, with Ascomycota being the most dominant (Fig. 2A; Fig. S1 and S3). In bat samples, the relative abundance of Basidiomycota was significantly higher in April compared to December and January (Kruskal−Wallis test: χ^2^ = 0.554, *P* = 0.002) (Fig. S1C). In cave environments, the relative abundance of Ascomycota was higher in far samples than in roost samples (Dunn’s test: Z = 3.33, *P* = 0.003) (Fig. S1A), while Mortierellomycota was significantly more abundant in roost samples compared to far samples (Dunn’s test: Z = −3.09, *P* = 0.006) (Fig. S1B).

At the genus level, significant changes in fungal composition were observed across hibernating bat, roost, and far samples (Fig. 2B; Fig. S2 and S4). Analysis of taxa with relative abundances greater than 0.5% ASVs showed that *Debaryomyces*, *Pseudogymnoascus*, and *Mortierella* were dominant in bat samples (Fig. S2B, C and E). In roost samples, the majority of ASVs were assigned to *Pseudogymnoascus*, *Mortierella*, and *Beauveria*, while in far samples, *Pseudogymnoascus* and *Beauveria* were the predominant genera (Fig. S2A through C). *Debaryomyces* populations were significantly more abundant in bat samples compared to roost and far samples (Kruskal−Wallis test: χ^2^ = 18.691, *P* < 0.001) (Fig. S2E). We identified 13 fungi belonging to psychrophilic fungi (Table S2). In addition, ANCOM analysis revealed that ASVs with varying abundances were present in both the time and site subgroups, but no significant variations were found in the infection status group (Table S3).

### Variation of fungal communities between bat skin and their hibernacula during hibernation

Generalized linear mixed models or linear mixed models, controlling for sampling time, revealed significant differences in alpha diversity among hibernating bat, roost, and far fungal communities, except for Evenness (Evenness: χ^2^ = 5.086, *P* = 0.079). Significant differences were found in Shannon diversity (χ^2^ = 9.279, *P* = 0.010) and the number of observed features (χ^2^ = 27.116, *P* < 0.001) (Fig. 2C through E; Fig. S5). Alpha diversity indices showed no significant differences among infection status groups (Fig. 2; Fig. S5C and F).

PCoA based on Bray-Curtis dissimilarity showed samples across the three sampling times during hibernation (Fig. 2F; Fig. S6A through C). Based on Bray-Curtis dissimilarity, dispersions were homogeneous across different groups (PERMDISP: F_time_ = 1.787, *P* = 0.171; F_site_ = 1.997, *P* = 0.140; F_infection_status_ = 1.020, *P* = 0.314) (Fig. S6D through F). Furthermore, we found that the beta diversity between the December and January samples was more similar to the April samples (Fig. S6G). PERMANOVA analysis revealed significant differences between sampling times and sample sites (F_time_ = 4.428, R^2^ = 0.059, *P* = 0.001; F_site_ = 5.815, R^2^ = 0.076, *P* = 0.001). Every month, significant differences were observed among bat, roost, and far samples (F_Dec_ = 2.195, R^2^ = 0.141, *P* = 0.001; F_Jan_ = 3.821, R^2^ = 0.116, *P* = 0.001; F_Apr_ = 3.227, R^2^ = 0.172, *P* = 0.001). No significant differences in beta diversity were observed between infected and uninfected samples (F_infection_status_ = 0.860, R^2^ = 0.006, *P* = 0.621).

### Processes of fungal community assembly in hibernating bats and their hibernacula

**Fig 3:**
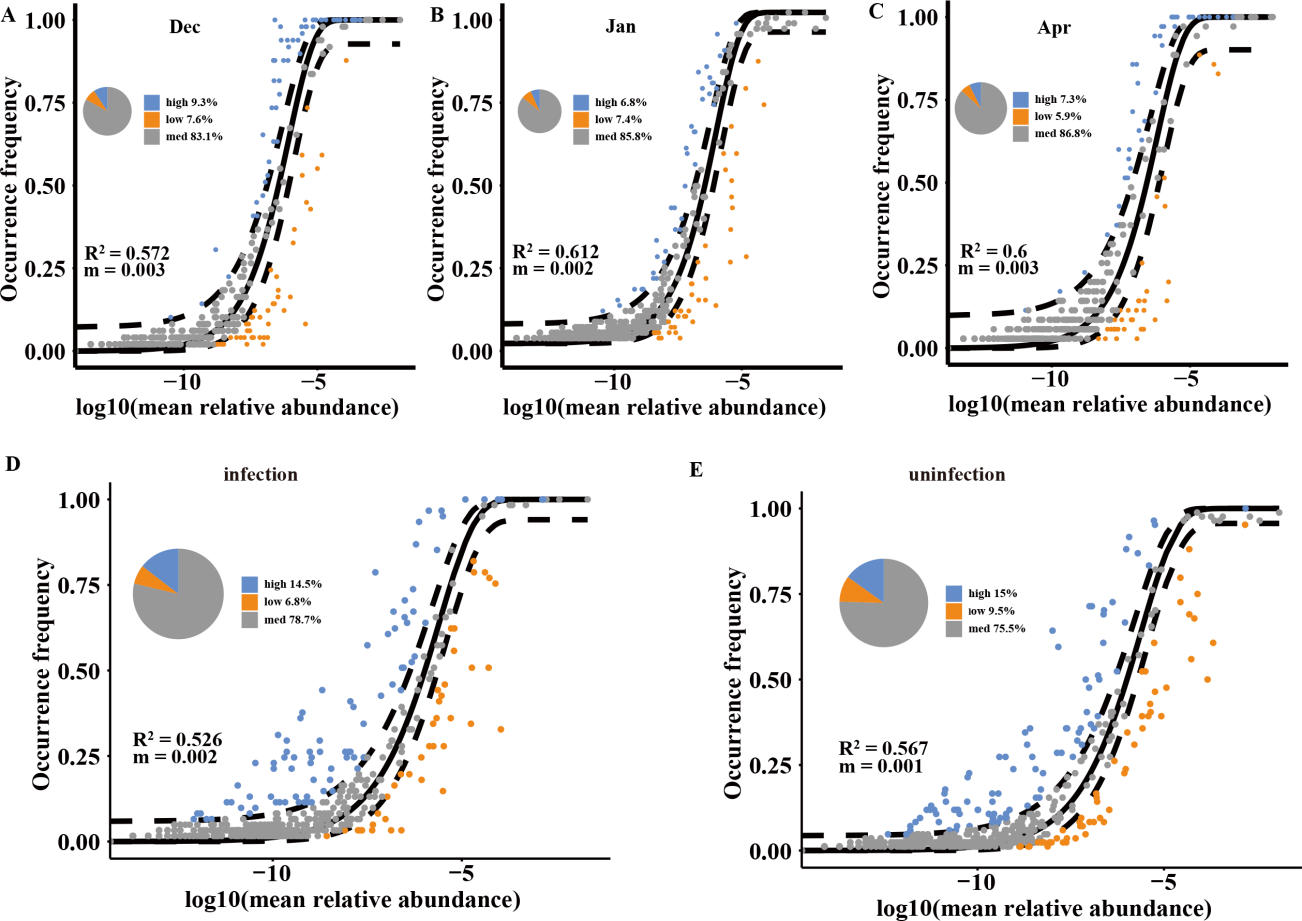
Neutral community model of fungal community assembly in hibernating bat, roost, and far samples at three times and in infected vs. uninfected samples during hibernation: (**A**) December 2017 (Dec), (**B**) January 2018 (Jan), (**C**) April 2018 (Apr), (**D**) infection, and (**E**) uninfection. Amplicon sequence variants (ASVs) that occur more or less frequently than predicted by the NCM are shown as blue or orange dots, respectively. Black solid lines indicate the 95% confidence interval around the prediction. Pie charts in each part of the figure represent the distribution of neutral processes, which occur more frequently than prediction (high), and occur less frequently than prediction (low) of ASVs in the hibernation period.

**Fig 4:**
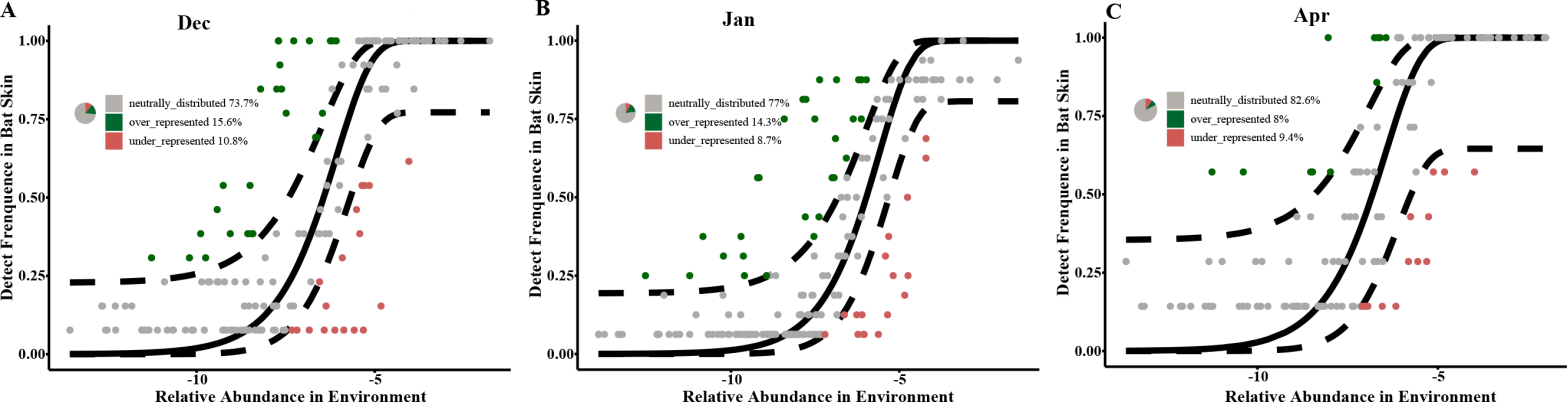
Implementation of the neutral model on *R. ferrumequinum* skin microbiota, using the environmental fungal reservoir as the source. ASVs that positively deviated from model expectations are represented by green points, while those that negatively deviated as shown as red points. Black solid lines indicate the 95% confidence intervals around the model predictions. The pie charts represent the relative importance of neutrally distributed, over-represented, and under-represented ASVs on bat skin in December (**A**), January (**B**), and April (**C**).

Fungal community assembly in hibernating bat, roost, and far samples was primarily driven by neutral processes, with the neutral community model showing strong fits (R^2^ = 0.572 to 0.612 (Fig. 3A through C). The dispersal impact, measured by the mobility (m), remains consistently low throughout the entire hibernation period, with minimal variation (0.001). As hibernation progressed, the proportion of ASVs influenced by neutral processes increased, from 83.1% in December to 86.8% in January (Fig. 3A through C). January bat and roost samples also conformed to the neutral model (R^2^ >0), but with more ASVs falling below predicted values (Fig. S7B and E). Both infected and uninfected groups adhered equally to the neutral model (Fig. 3D and E). Our study found that 73.7%–82.6% of the fungal communities on bat skin showed a neutral distribution pattern in December, January, and April, with the cave environments acting as the source of the fungal community on bat skin (Fig. 4). Specifically, in December, 15.6% ASVs were over-represented and 10.8% were under-represented; in January, these percentages were 14.3% and 8.7%, respectively, and in April, they were 8% and 9.4%, respectively (Fig. 4; Table 1).

**TABLE 1:** Number of ASVs on bat skin that were consistently over-represented or under-represented

	Total ASVs	Neutral distribution	Over-represented	Under-represented
December	167	123	26	18
January	196	151	28	17
April	138	114	11	13

**Fig 5:**
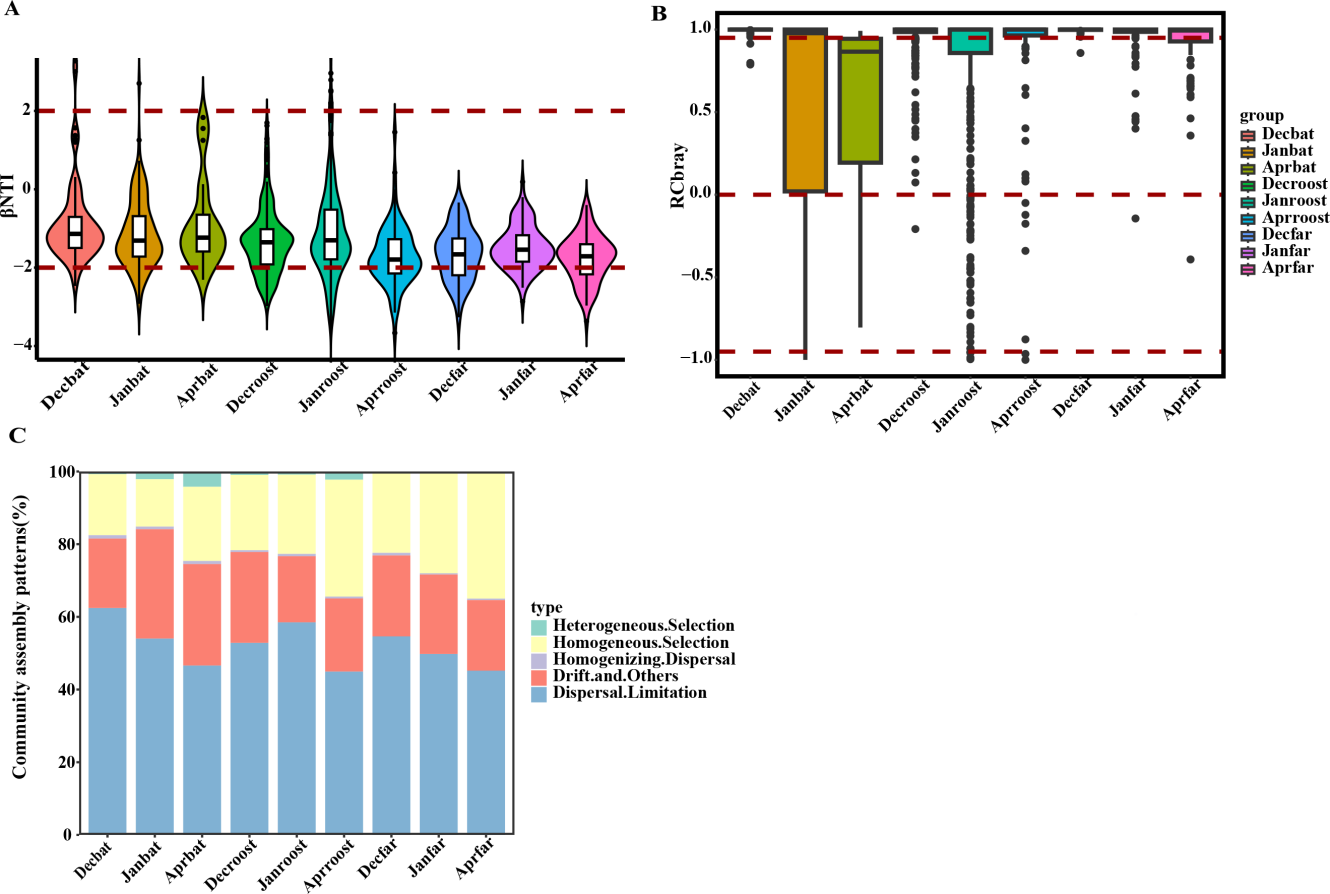
Community assembly patterns of fungal communities in hibernating bat, roost, and far samples: (**A**) β Nearest Taxon Index (βNTI) distribution; red dashed lines for βNTI values of −2 and 2; (**B**) box plots of Raup−Crick (RCbray) values calculated by the Bray−Curtis method, with dashed lines indicating RCbray values of 0.95, 0, and −0.95; (**C**) percentage of fungal community assemblies that were primarily controlled by various deterministic factors (including homogeneous and heterogeneous selection) and stochastic processes (including dispersal limitation and homogenizing dispersal) as well as the percentage of fungal community assembly controlled by drift processes.

Ecological processes influencing fungal community assembly on bat, roost, and far samples were assessed using null model tests, focusing on phylogenetic and taxonomic diversity indices (βNTI and RCbray). The results showed that most βNTI values (78.98%) across all samples were between −2 and 2, indicating that stochastic processes primarily governed fungal community dynamics (Fig. 5A). In addition, 74.07% of RCbray values exceeded 0.95, highlighting the significant role of dispersal limitation in shaping these communities (Fig. 5B). Dispersal limitation and drift were dominant processes in bat samples, whereas roost and far samples were more influenced by dispersal limitation and homogeneous selection (Fig. 5C). Overall, these findings suggest that fungal community assembly in bat, roost, and far samples is driven by a combination of stochastic and deterministic processes, with stochastic processes being the predominant influence.

## DISCUSSION

In this study, we investigated the fungal community composition, diversity, and assembly in hibernating *R. ferrumequinum* and their roost cave environments in the presence of the fungal pathogen *P. destructans*. The relative abundance of Ascomycota was high both on the bat skin and in the hibernaculum (45). Recent studies on bat skin and hibernation cave fungal communities have reported similar results (45–48). Ascomycota exhibits a wide range of life modes, including pathogenic (agriculturally and clinically), saprobic, and endophytic, and has been extensively studied (49). Ascomycota are dominant in cave environments, regardless of the presence of WNS (50, 51). Several fungal genera within Ascomycota, including *Alternaria* (48, 51, 52), *Penicillium* (48, 51, 53–55), *Gymnoascus* (56), *Pseudogymnoascus* (46, 52, 57), *Beauveria* (58), *Debaryomyces* (46, 59, 60), and *Oidiodendron* (47, 51, 56) are commonly found on bat skin and in cave environments in recent research. The widespread presence of these fungal taxa across multiple caves and bat species indicates their potentially significant role in these environments.

*Pseudogymnoascus*, a psychrophilic genus, was detected both in cave environments and on bats’ skin. Notably, it colonizes bat skin and can persist independently in the environments (24). Research on fungal communities in American bat hibernacula has shown that these sites can host various nonpathogenic *Pseudogymnoascus* species (3, 61). These fungi are typically found in low-temperature environments, thriving even in Antarctic alpine perennial permafrost at temperatures as low as −5°C (62). Their resilience in cold habitats may be due to specialized physiological and metabolic adaptations, as well as unique secondary metabolites. *Pseudogymnoascus destructans* loads and prevalence remained consistently low on both bat skin surfaces and in the surrounding environments throughout the hibernation period, suggesting a degree of resistance in bats against *P. destructans* infection. Host microbiota are crucial in protecting the host from foreign and potentially pathogenic microorganisms, with fungal microbial communities playing a significant role in these defense mechanisms (12). Certain host-associated taxa can produce antimicrobial compounds that inhibit or eliminate potential invaders, thereby protecting both the host and the associated taxa (16). Moreover, some host microorganisms may be acquired from the environments (63), highlighting the intricate relationships between host, environment, and microbial defense systems.

The fungal taxa detected on bat bodies and in cave environments in this study may display potential antifungal properties. For instance, *Alternaria* taxa have shown resistance to pathogenic bacteria by inducing cell death through the production of toxic substances (64). Notably, *Debaryomyces* was found in much higher relative abundance on bat skin compared to the environments, over 20 times greater, and has been shown to completely inhibit *P. destructans* growth under certain conditions (59). Although not highly abundant, the genera *Penicillium*, *Simplicillium*, and *Aureobasidium* fungi found on bat skin and in the environments exhibit inhibitory properties against plant bacterial and fungal diseases (65–68), underscoring the potential role of these fungal taxa in bat defense mechanisms (60).

A study conducted in North America is consistent with our research observations (45), showing that the alpha diversity of the fungal community in hibernating *R. ferrumequinum* was significantly lower than that in the surrounding cave environments. Compared to external environments, cave environments are often regarded as very stable and relatively homogeneous (69). Many caves maintain relatively constant high humidity levels, making them ideal for a variety of microorganisms. These environments serve as natural reservoirs for fungi, including those adapted to specific conditions such as psychrophilic (70), thermophilic (71), basophilic (72), and halophilic (73) species, which acquire additional microorganisms from environmental substrates. In the community assembly processes of fungal communities on bat skin and cave environments, stochastic processes dominate. Fungi disperse from the environments, influencing the presence and relative abundance of microorganisms, which ultimately leads to changes in alpha diversity at different sampling times and across different sites (39). The sampling site is an important factor influencing the overall diversity and composition of the skin fungal community (59) and host skin microbial communities are mainly derived from local environments (17). The cave microenvironments, including factors like temperature, experiences constant changes throughout the hibernation period. Bats can lower their body temperature to match the ambient temperature around the roosting samples, which could significantly impact the fungal community structure. PERMANOVA analysis also revealed significant variations in sampling times. During the pre-hibernation period, bats may experience a transitional phase where their fungal communities likely undergo restructuring and specialization to adapt to the physiological and behavioral changes associated with hibernation. The skin microbiota of bats might play a crucial role in defending against *P. destructans* infection, potentially intensifying as hibernation progresses to enhance protection against pathogens (74). This phenomenon also helps explain the variation in fungal communities on bat skin and environments with increasing hibernation duration. However, there were no significant differences in *P. destructans* infection status between bat, roost, and far samples during hibernation, despite significant differences in both alpha and beta diversity. The PCoA visualization did not reveal distinct temporal clustering. This may be due to the substantial overlap of species across different time points, leading to high similarity between samples despite the statistical significance observed in the PERMANOVA tests. In addition, other environmental variables not accounted for in the analysis may have influenced the community composition, contributing to the observed outcomes. In addition, ANCOM analysis showed no differential species in the fungal communities across different infection status (Table S3). One potential explanation for this result is the relatively low loads of *P. destructans* infection in Chinese bats and their hibernacula, coupled with the absence of apparent symptoms in the bats, which may not have caused notable variations in the fungal community between cave environments and bat skin.

Our results underscore the prominent role of stochastic processes in shaping fungal community assembly. Related studies have also highlighted the importance of these processes in fungal community development (29, 75–78). This finding suggests that stochastic processes have a stronger influence compared to deterministic factors. This observation is consistent with similar findings regarding bacterial community assembly on bat body surfaces (79). ASVs that diverge from the neutral community model may represent species undergoing environmental selection or microbes with distinct dispersal capabilities compared to those in the surrounding environments (39). Research has found that under-represented ASVs may lead to low dispersal potential (39). *P. destructans* as a fungal pathogen, understanding the assembly process of ASVs within the *Pseudogymnoascus* is essential for evaluating the pathogen’s transmission potential. Our findings indicate that ASVs within the *Pseudogymnoascus* exhibit neutral or underrepresentation, suggesting a limited transmission ability of *P. destructans* from the environment to bat skin. This may help explain why *P. destructans* loads are not significantly correlated between bats and the environments. The m-value, a metric for community-level mobility, indicates restricted species dispersal within the community when low. The fungal communities in hibernating bat, roost, and far samples displayed notably limited dispersal abilities. This pattern was consistently reflected in analyses of community development processes using metrics such as βNTI and RCbray values.

Dispersal limitation emerged as the predominant assembly process across all sample groups, exceeding 40% in each case. Restricted movement or survival of organisms in new locations can lead to significant diversity between communities (27). Bats hibernate in relatively closed cave ecosystems, which may restrict fungal dispersal (80). Unlike bacteria, fungi are mainly spread via spores, leading to lower dispersal capacities and limited exchange between populations, thus affecting community turnover (81). The presence of dormant stages and lower growth rates during bat hibernation in cooler environments further influences community assembly. The relative abundance of microbial operational taxonomic units is linked to processes like homogenous selection, ecological drift, and dispersal limitation, as described by Ma et al. (76). Ambient temperatures may favor the dispersal of certain cryophilic fungi while restricting others, impacting community composition. Our results also show significant homogeneous selection across subgroups, indicating that consistent abiotic and biotic conditions contribute to structural similarity among communities (27). Previous research suggests that both homogeneous selection and dispersal contribute to community structure homogenization (82). The high degree of homogeneous selection observed in our study is likely due to the consistent environmental conditions at our sites.

This study highlights that neutral processes dominate the fungal community assembly on bat skin and in the roost cave environments and that the fungal community in both environments may inhibit fungal growth. However, due to limitations in the sequencing methods used, we were unable to definitively identify species at the species level. In addition, we cannot confirm whether these potential anti-*P. destructans* fungal taxa actually contribute to inhibition. Future work will involve isolating and validating anti-*P. destructans* fungi from bat skin and roosting cave environments through media culture experiments. Furthermore, metagenomic sequencing will be employed to elucidate the specific functions of these species. This will contribute to a better understanding of the role of fungal community assembly processes in the skin and surrounding environment microbiota.

## MATERIALS AND METHODS

### Field sampling

A total of 37 skin swabs from adult *R. ferrumequinum* and 107 swabs from cave rock surfaces were collected at Gezi Cave, Jilin Province, China, across three time points: December 2017, January 2018, and April 2018 (Table 2).

**TABLE 2:** Information from bat skin and cave environment samples fungal microbiota

Sampling date	Bat skin samples (uninfected/infected)	Roost samples (uninfected/infected)	Far samples (uninfected/infected)
December 4, 2017	13 (10/3)	24 (16/8)	11 (7/4)
January 18, 2018	17 (10/7)	30 (12/18)	14 (7/7)
April 9, 2018	7 (4/3)	14 (11/3)	14 (6/8)

Gezi cave is a limestone cave with a 1.8 km length and a 48 m altitude drop, descending toward the entrance. Long-term surveys show a stable population of about 300 *R. ferrumequinum*, hibernating from October to April. The cave temperature averages between 3.27°C and 6.92°C.

Microbial samples were collected from three sites: (i) bat skin swabs (bat sample), (ii) the environment within 10 cm of a hibernating bat (roost sample), and (iii) random sites on the cave wall, ranging from the entrance to the innermost part of the cave (far sample). The distance between any two sites exceeds 2 m, and no bats roost these areas.

Bat skin swabs were collected by wiping the muzzle and forearm sections of each bat five times with a sterile cotton swab dipped in sterile water. Each bat was carefully removed from the roost using a pair of sterile latex gloves and swabbed with sterile polyester swabs. Each swab was then placed in a sterile tube with 500 µL of RNAlater (Tiangen Biotech Co., Ltd., Beijing, China) (83). Each bat was released after sampling. For cave environments (roost and far samples), a dry and sterile cotton swab was rubbed across the rock surface five times at each site. Samples were taken from different bats at random, with far samples collected at fixed locations over three time points. All samples were preserved at −80°C for genomic DNA extraction. DNA extraction from all samples collected from the three collection periods was performed simultaneously.

### Fungal DNA extraction, detection, and sequencing

Genomic DNA was extracted from bat skin and cave swabs using DNeasy blood and tissue extraction kits (Qiagen, Hilden, Germany), with negative controls included to check for contamination (83). After DNA extraction, samples were tested for *P. destructans* using fluorescent quantitative PCR (qPCR) on a Stepone instrument (Applied Biosystems, USA), following Muller et al.’s method (61). Each sample was analyzed in duplicate for accuracy, with positive controls from *P. destructans* ATCC MYA-4855. To calculate the fungal load, a positive control quantitative standard (2 ng/µL) was designed with the formula: log10(*Pd* (ng)) =(Ct − 22.04942)/−3.34789) (83). None of the negative controls detected contamination, and a sample was deemed positive for *P. destructans* infection if its Ct value was detected (i.e., if it was below the maximum threshold of 50).

PCR amplification utilized genomic DNA as a template and the resulting products underwent subsequent amplicon sequencing. The extracted genomic DNA was accurately quantified using the Qubit 3.0 DNA detection kit (ThermoFisher Scientific, Wilmington, DE, USA) to determine the appropriate amount for the PCR. The primers ITS1F (CTTGGTCATTTAGAGGAAGTAA) and ITS2R (GCTGCGTTCTTCATCGATGC) targeted the ITS1-ITS2 region of fungal genomic DNA (84). We performed two rounds of PCR amplification to increase the yield of amplification products. The amplification conditions were as follows: pre-denaturation at 94°C for 3 min; five cycles of denaturation at 94°C for 30 s, re-denaturation at 45°C for 20 s, extension at 65°C for 30 s; followed by 20 cycles of denaturation at 94°C for 20 s, annealing at 55°C for 20 s, extension at 72°C for 30 s; and final extension at 72°C for 5 min. Subsequently, a second round of amplification was performed using the obtained PCR products. The conditions for this round were as follows: pre-denaturation at 95°C for 5 min; five cycles of denaturation at 94°C for 20 s, re-denaturation at 55°C for 20 s, extension at 72°C for 30 s, and finally extension at 72°C for 5 min. To ensure high-quality sequencing data, the concentration of the libraries was determined and purified using the Qubit 3.0 fluorescence quantifier according to the manufacturer’s instructions. Sequencing was conducted with paired-ended reads of 2 × 300 bp, using the Illumina Miseq platform at Shanghai Sangon Biotech Co., Ltd. (Shanghai, China). To prevent batch effects, all samples were sequenced in a single batch.

The raw data were imported into QIIME2 (version 2020.08) (85), and quality filtering and denoising were performed using DADA2 (--p-trunc-len 237), which generated an ASV table and representative sequences (86). Chloroplasts, mitochondria, and ASVs present in fewer than two individuals were removed. Taxonomic classification was conducted using the classify-sklearn method in the feature-plugin for QIIME2 with the unite-ver8-99-classifier-04.02.2020 database and the Warcup Fungal ITS Trainset 2 RDP database (75, 87–89), covering multiple taxonomic levels (kingdom, phylum, order, order, family, genus, and species) (90). Results from both databases were integrated to enhance species identification accuracy. In cases of database inconsistency, a higher level of identification was adopted; if conflicting results were provided at the same level, the consistent taxonomic level was retained. Phylogenetic trees were constructed using the align-to-tree-mafft-fasttree method in the phylogeny plugin for QIIME2 (91). The effect of different groups (Time: Dec, Jan, Apr; Site: bat, roost, far; Infection status: infection, uninfection) on microbial taxa composition down to the genus level was assessed using the Analysis of Composition of Microbiomes (ANCOM 2.0) (92) base on composition add-pseudocount (--p-pseudocount 1) and composition ancom plugins for QIIME2. A higher W value indicates a more pronounced difference in features. Relative abundance >0.5% ASV table was analyzed, and the table was rarefied (-p--depth 2692). After merging, quality control, and filtering, a total of 10,376,366 validated reads were obtained with an average read length of 237 bp. Following the removal of amplicon sequence variants (ASVs) present in only two individuals, 592 ASVs were retained for subsequent analyses.

### Data analysis

The prevalence and loads of *P. destructans* infection on bat, roost, and far samples over time were compared using a nonparametric Kruskal–Wallis test. Dunn’s multiple comparisons test with Bonferroni correction was then applied using the FSA package (93). We applied a logarithmic modification to the *P. destructans* load values for visualization, due to their low magnitude.

To elucidate the dynamics of the fungal communities on the skin of hibernating bats, as well as in roost and far samples, we analyzed the data at both phylum and genus taxonomic levels (relative abundance >0.5%). Variations in fungal communities across bat, roost, and far samples at these levels were compared using the dunntest() function in the FSA package, with corrections applied using the Bonferroni method (93). Simultaneously, we also searched for fungi with psychrophilic and psychrotrophic characteristics through a literature search (94).

To elucidate the variation in fungal communities across different groups (Time: Dec, Jan, Apr; Site: bat, roost, far; Infection status: infection, uninfection), several alpha diversity indices, including Shannon diversity, number of observed features, and Evenness were calculated for each sample. The core-metrics-phylogenetic command, with a sampling depth of 2,936, was used in the diversity plugin within QIIME2 to compare overall alpha diversity, richness, and evenness. Generalized linear mixed models or linear mixed models compared alpha diversity among groups, with significance tested using the Anova() function of the car package in R, considering sampling time as a random effect. Variations between time and site groups were assessed using a nonparametric Kruskal–Wallis test, with Bonferroni correction for multiple comparisons. To clarify the differences in alpha diversity between the infected and uninfected groups, we used the wilcox.test() function from the stats package for testing.

The Bray–Curtis dissimilarity was computed using the q2-beta diversity plugin in QIIME2. This metric was then analyzed through principal coordinate analysis (PCoA) using the cmdscale() function from the vegan package in R to visualize sample dissimilarities (95). The permutational analysis of multivariate dispersions (PERMDISP) used betadisper() in the vegan package to evaluate the homogeneity of dispersion among sample groups. Furthermore, we compared the differences in average dispersion between groups of time, site, and infection status through Tukey’s test (96). To assess differences in beta diversity among fungal communities across different groups, a permutation multivariate analysis of variance (PERMANOVA) with 999 permutations was performed using the adonis2() function of the vegan package in R (97). To clarify the similarity of beta diversity between samples collected at different time points, we used the vegan package to calculate the Bray–Curtis dissimilarity among distinct sampling points (98). To assess the overall difference among time points, we conducted a Kruskal–Wallis test. Following the detection of significant overall differences, we performed pairwise comparisons using the Wilcoxon rank-sum test to further investigate the beta diversity variations between the different sampling time points.

To assess the processes shaping fungal communities on and around hibernating bats, several analyses were conducted. First, Neutral community models (NCM) of fungal community assembly in hibernating bat, roost, and far samples at three time points during hibernation were applied using the neutral.fit() function from the MicEco package in R (99). The goodness of fit of the neutral model to fungal communities was assessed using the coefficient of determination (R^2^), and the estimated migration rate was represented with the parameter m. ASVs were categorized based on their occurrence relative to the 95% confidence interval of NCM predictions into above, below, or neutral partitions. We considered the fungal communities in the environments (roost and far samples) as the source of fungi on bat skin, using the Hmisc package to quantify the neutral and selective processes shared between bat skin and the environments for ASVs in R. ASVs were classified into three parts, neutrally distributed, over-represented (positive selection distribution), and under-represented (negative selection distribution) (79, 100). In addition, β Nearest Taxon Index (βNTI) and Raup–Crick (RCbray) index were calculated, with null distributions constructed using the qpen() function in the iCAMP package, with thresholds set at |βNTI| = 2 and |RCbray| = 0.95, and randomized 1,000 times. βNTI values > 2 or < −2 indicated deterministic processes of heterogeneous or homogeneous selection, while values between −2 and 2 suggested stochastic processes. RCbray values < −0.95 or >0.95 indicated significant compositional differences, with values between −0.95 and 0.95 suggesting drift (31, 99). Kruskal−Wallis tests were used to examine differences in βNTI and RCbray indices among hibernating bat, roost, and far samples (30, 101). All analyses and visualizations were performed using R version 4.3.3.

## Data Availability

The raw sequence data are submitted to the National Center for Biotechnology Information (NCBI) Sequence Read Archive under accession number PRJNA781296.

## SUPPLEMENTAL MATERIAL

Figure S1 spectrum.02233-24-s0001.tif Relative abundance at the phylum level.

Figure S2 spectrum.02233-24-s0002.tif Relative abundance at the genus level.

Figure S3 spectrum.02233-24-s0003.tif Bar chart of relative abundance of phylum level.

Figure S4 spectrum.02233-24-s0004.tif Bar chart of relative abundance of genus level.

Figure S5 spectrum.02233-24-s0005.tif Fungal community alpha diversity.

Figure S6 spectrum.02233-24-s0006.tif Beta diversity figures of samples.

Figure S7 spectrum.02233-24-s0007.tif Neutral community model of fungal community assembly of samples.

Supplemental material spectrum.02233-24-s0008.pdf Supplemental figure legends and Tables S1 to S3.

ASM does not own the copyrights to Supplemental Material that may be linked to, or accessed through, an article. The authors have granted ASM a non-exclusive, world-wide license to publish the Supplemental Material files. Please contact the corresponding author directly for reuse.
