# Supplementary figures and images for: Variation and assembly mechanisms of Rhinolophus ferrumequinum skin and cave environmental fungal communities during hibernation periods
Source: Microbiol Spectr. 2025 Jan 23;13(3):e02233-24. doi: 10.1128/spectrum.02233-24 (PMC11878040; doi:10.1128/spectrum.02233-24)

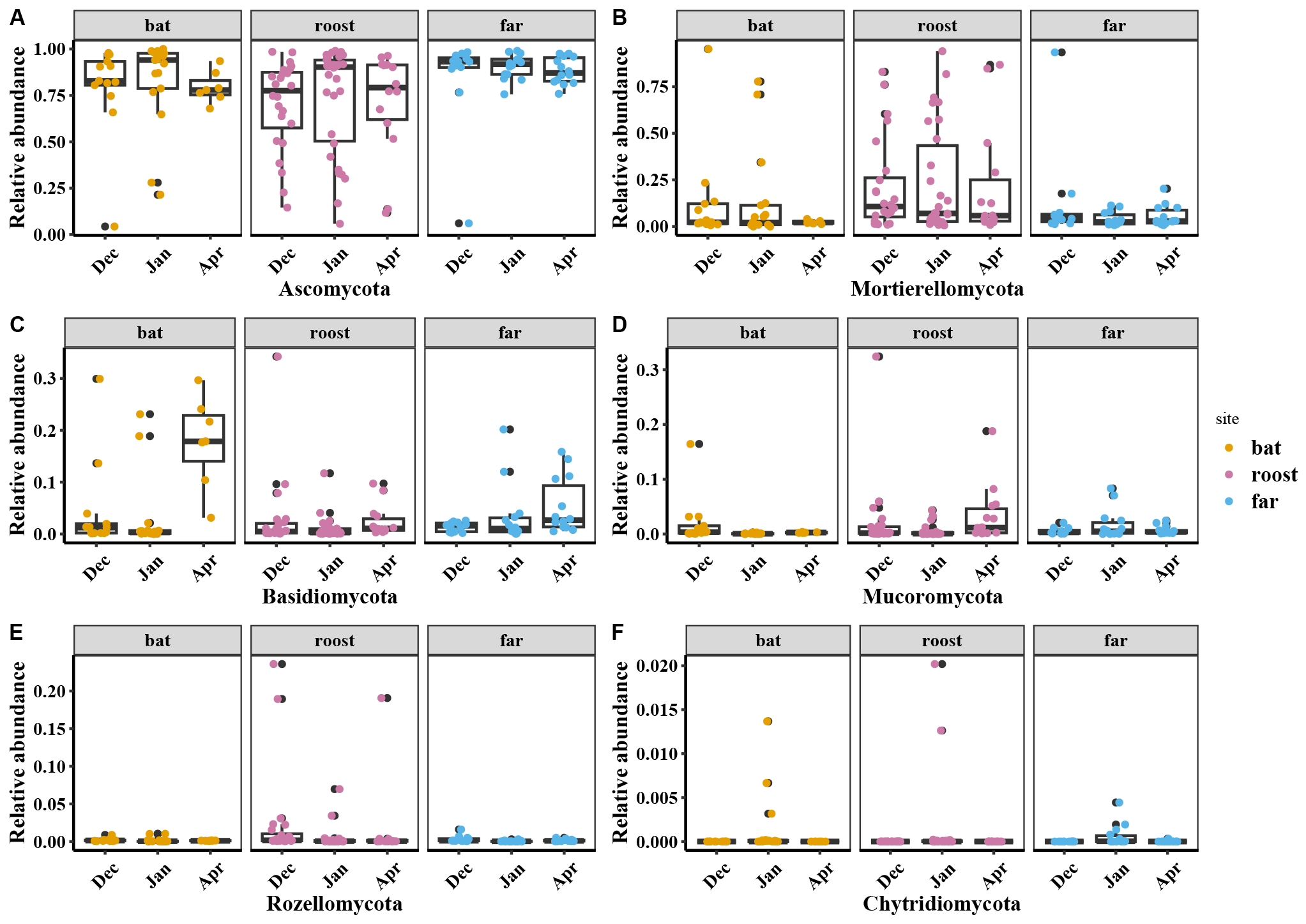

Supplement: Figure S1 — Relative abundance at the phylum level. [file spectrum.02233-24-s0001.tif]

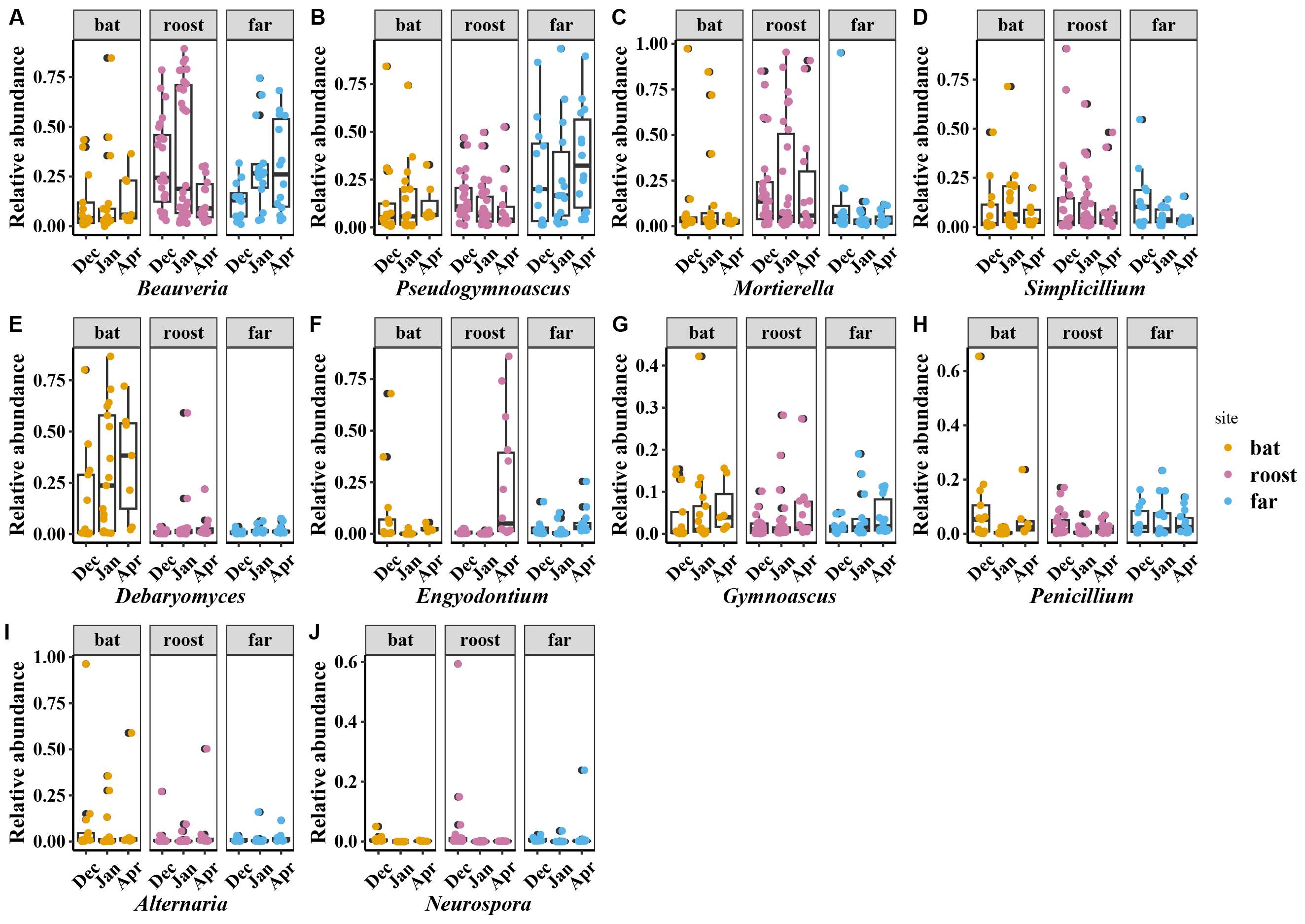

Supplement: Figure S2 — Relative abundance at the genus level. [file spectrum.02233-24-s0002.tif]

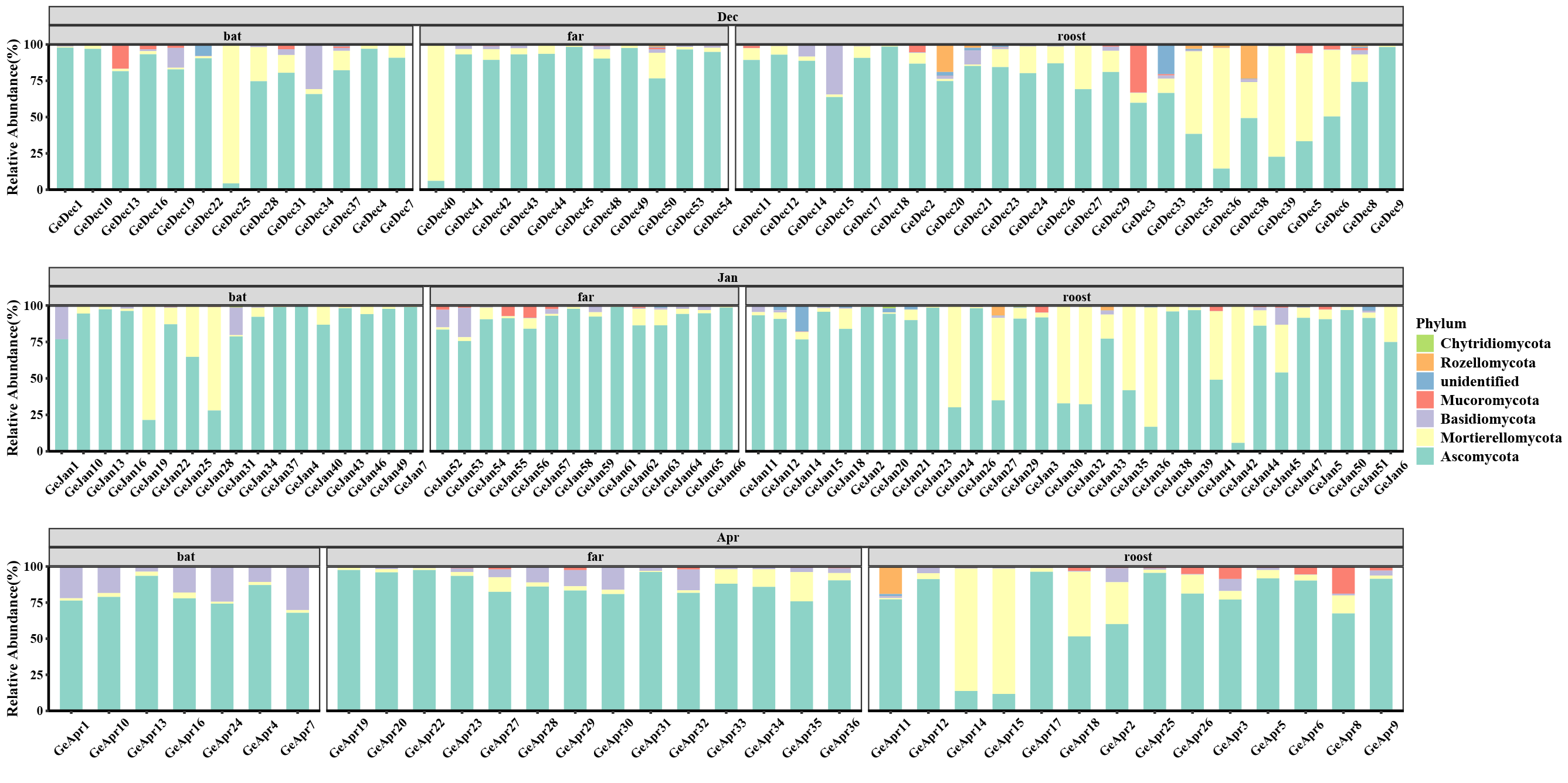

Supplement: Figure S3 — Bar chart of relative abundance of phylum level. [file spectrum.02233-24-s0003.tif]

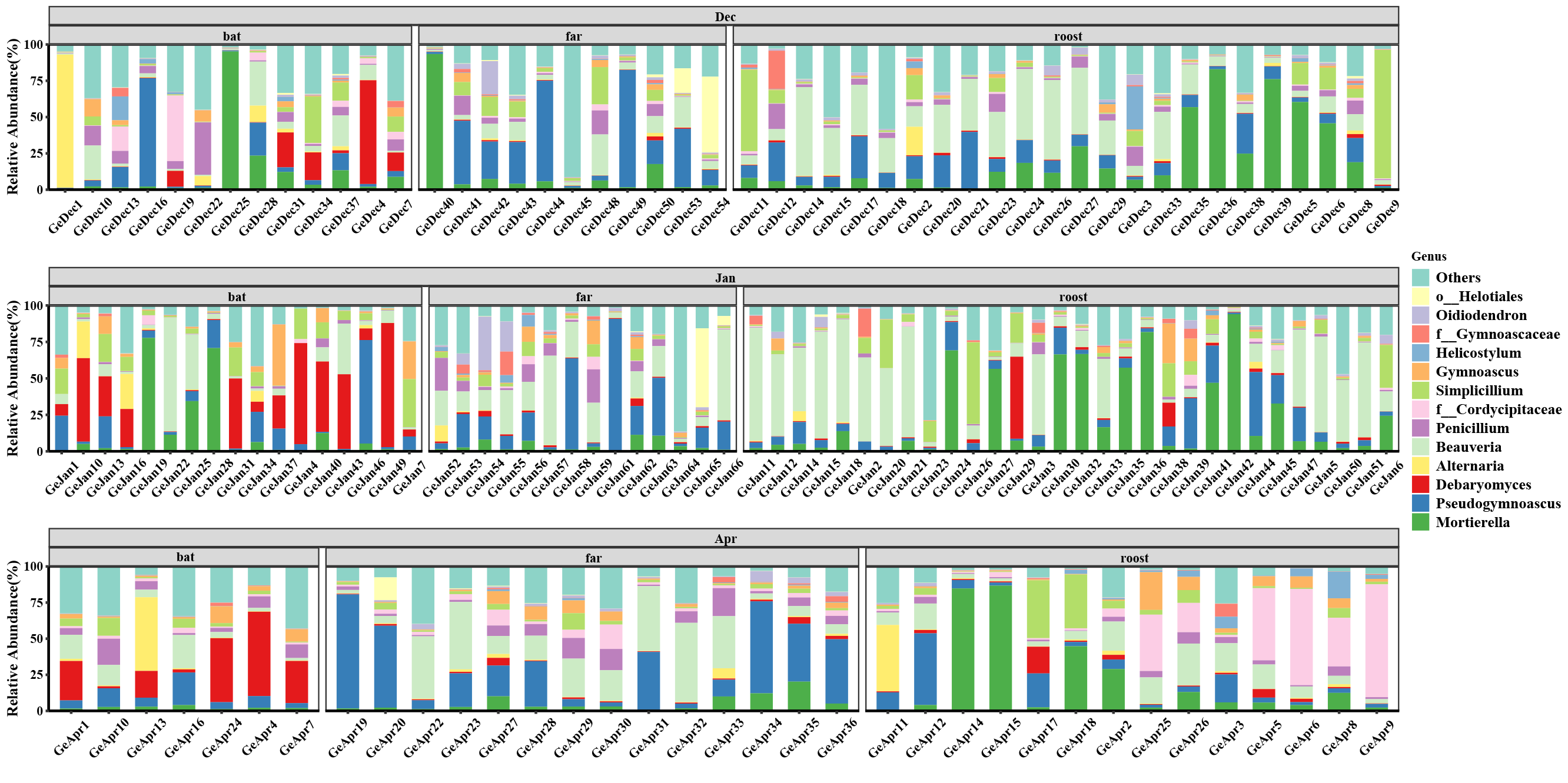

Supplement: Figure S4 — Bar chart of relative abundance of genus level. [file spectrum.02233-24-s0004.tif]

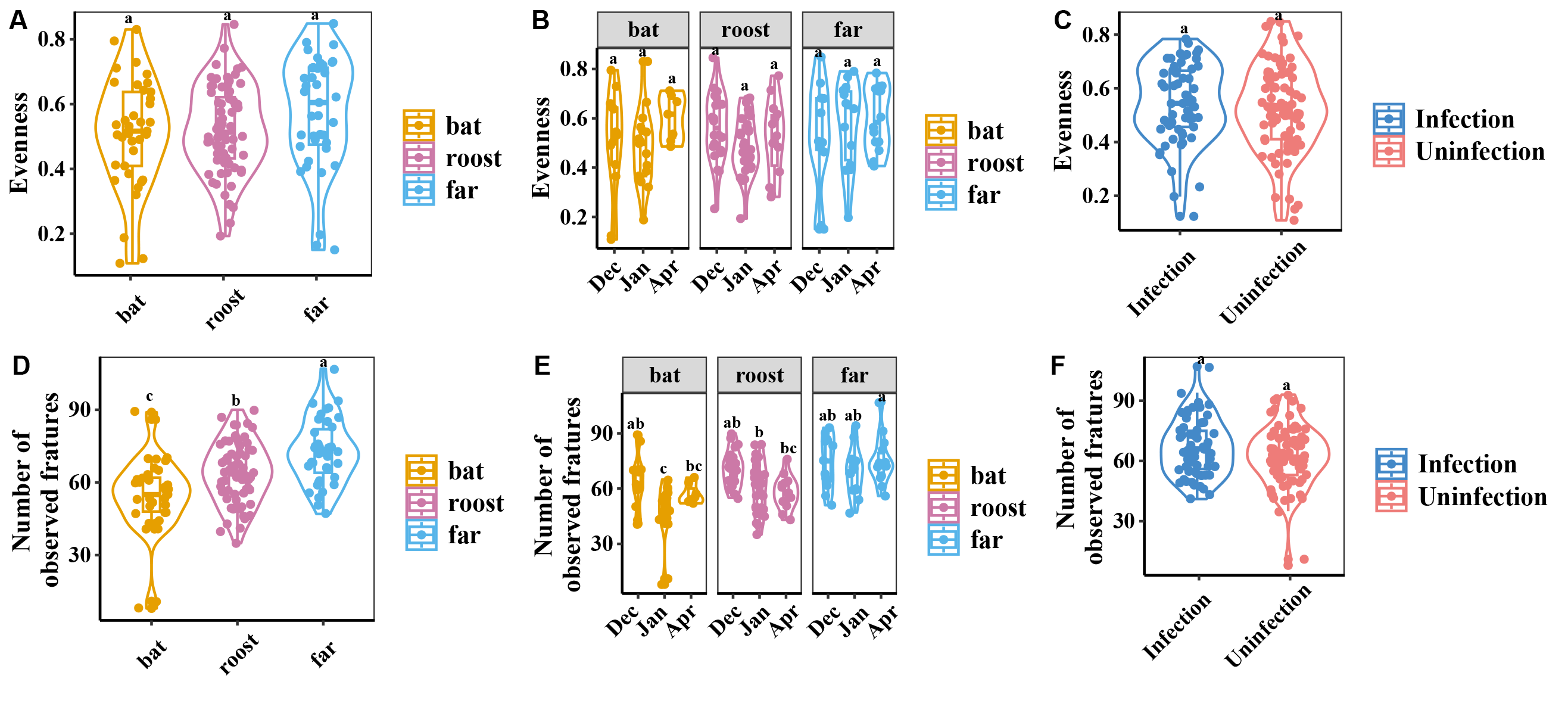

Supplement: Figure S5 — Fungal community alpha diversity. [file spectrum.02233-24-s0005.tif]

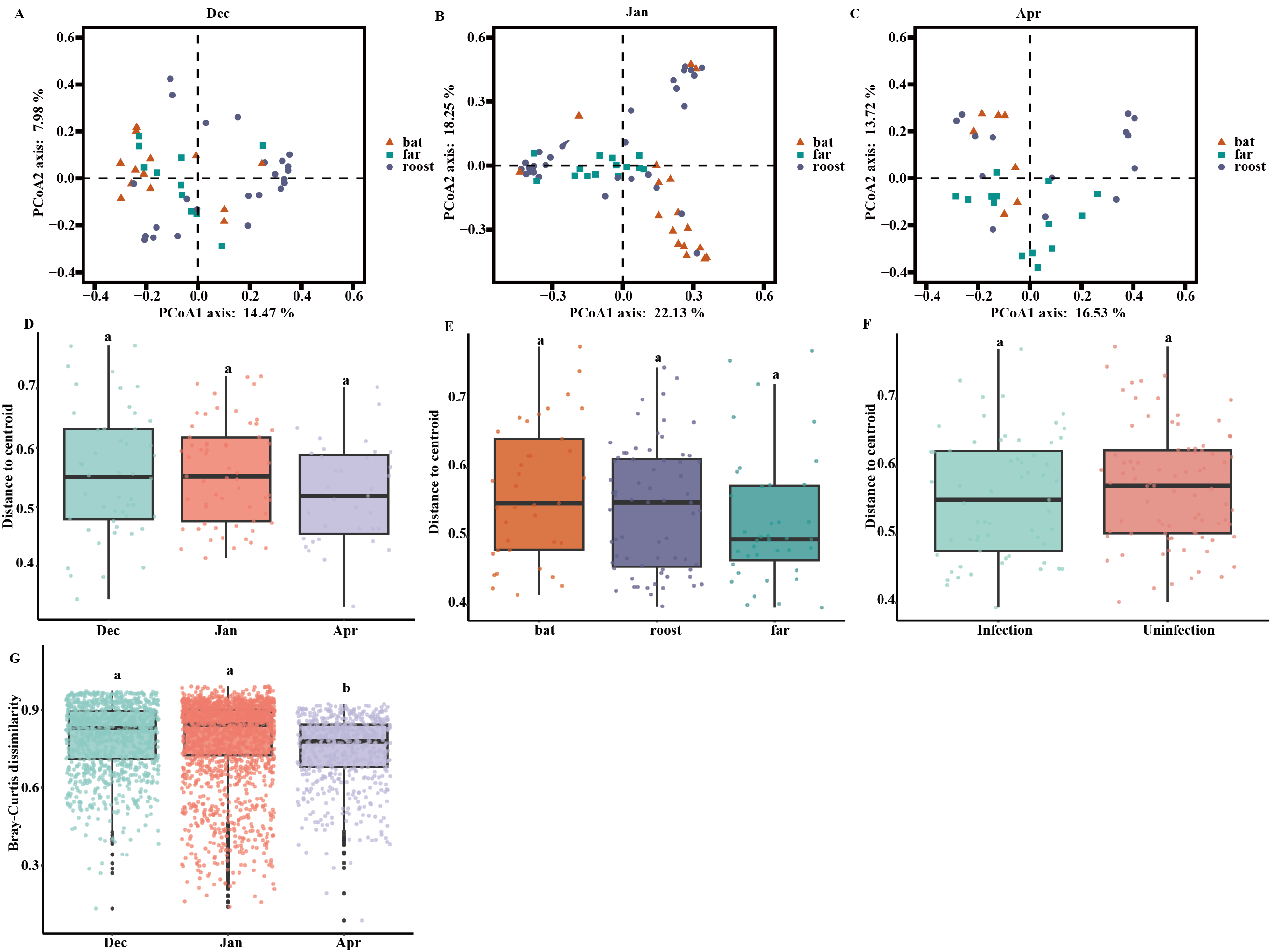

Supplement: Figure S6 — Beta diversity figures of samples. [file spectrum.02233-24-s0006.tif]

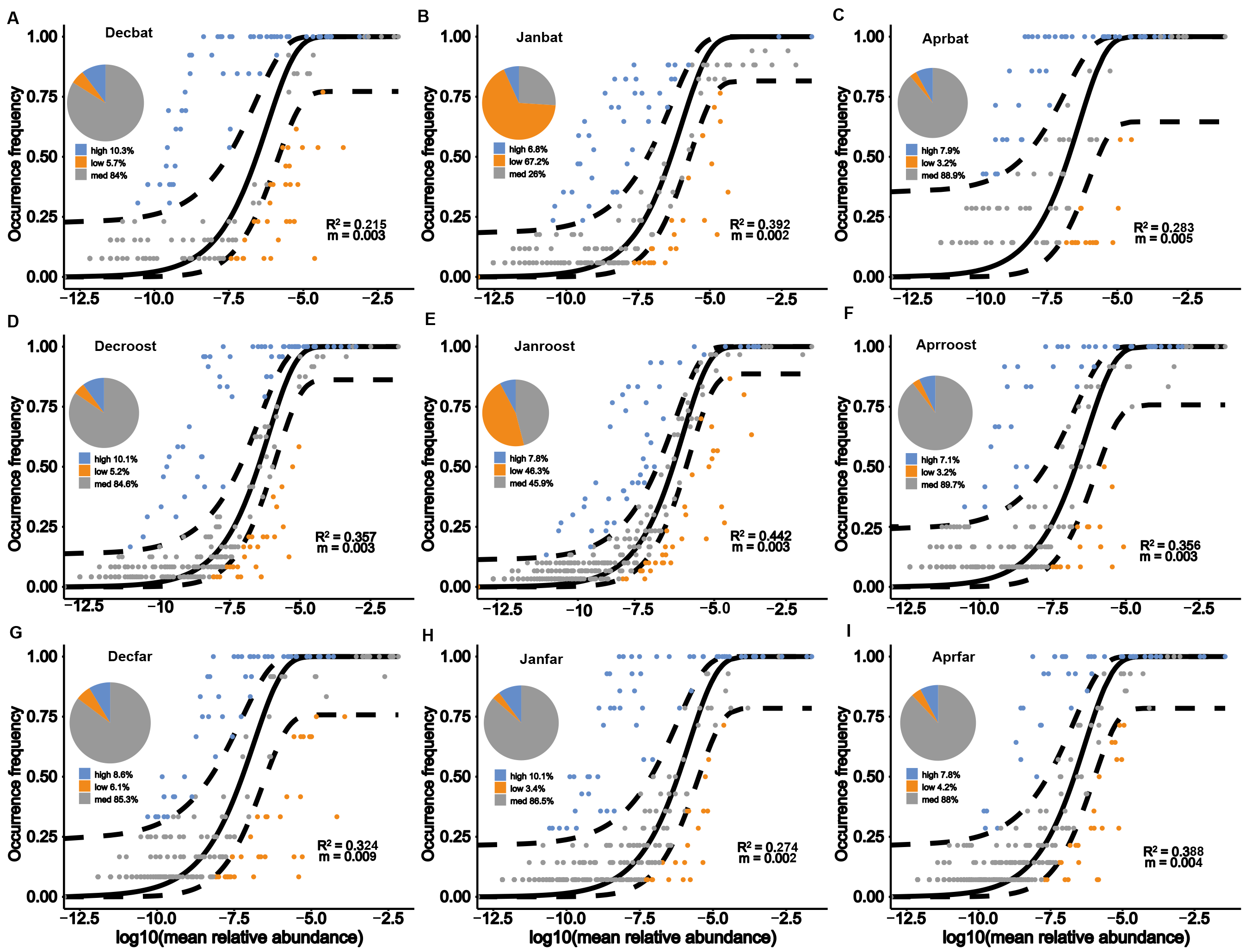

Supplement: Figure S7 — Neutral community model of fungal community assembly of samples. [file spectrum.02233-24-s0007.tif]
