# Supplementary material for: Variation and assembly mechanisms of Rhinolophus ferrumequinum skin and cave environmental fungal communities during hibernation periods
Source: Microbiol Spectr. 2025 Jan 23;13(3):e02233-24. doi: 10.1128/spectrum.02233-24 (PMC11878040; doi:10.1128/spectrum.02233-24)
Supplement: Supplemental material — Supplemental figure legends and Tables S1 to S3. [file spectrum.02233-24-s0008.pdf]

Supplemental Materials

Supplemental Figure Legends

FIG. S1 Relative abundance of bat, roost, and far fungal communities at the phylum level during the hibernation period during December 2017 (Dec), January 2018 (Jan), and April 2018 (Apr): (A) Ascomycota, (B) Mortierellomycota, (C) Basidiomycota, (D) Mucoromycota, (E) Rozellomycota, (F) Chytridiomycota.

FIG. S2 Relative abundance of bat, roost, and far fungal communities at the genus level during hibernation at December 2017 (Dec), January 2018 (Jan), and April 2018 (Apr) (Overall relative abundance >0.5%): (A) *Beauveria*, (B) *Pseudogymnoascus*, (C) *Mortierella*, (D) *Simplicillium*, (E) *Debaryomyces*, (F) *Engyodontium*, (G) *Gymnoascus*, (H) *Penicillium*, (I) *Alternaria*, (J) *Neurospora*.

FIG. S3 Bar chart of relative abundance of phylum level for samples.

FIG. S4 Bar chart of relative abundance of genus level for samples (relative abundance > 0.5%).

FIG. S5 Fungal community alpha diversity (Evenness and Number of observed features) during hibernation: (A) fungal community Evenness among bat, roost, and far samples; (B) fungal community Evenness among December, January, and April; (C) fungal community Evenness between different infection status; (D) fungal community number of observed

features among bat, roost, and far samples; (E) fungal community number of observed features among December, January, and April; (F) fungal community number of observed features between different infection status.

FIG. S6 Graph of principal coordinate analysis (PCoA) of Bray-Curtis dissimilarity for (A) December, (B) January, and (C) April; box plot of multivariate homogeneity of group dispersions (variances) of (D) December, January, April, (E) bat, roost, far, (F) infection and uninfection; and (G) beta-diversity analsis across December, January and April based on Bray–Curtis dissimilarity. Data points that do not share a letter are considered significantly different between treatments.

FIG. S7 Neutral community model of fungal community assembly in bat, roost, and far samples during hibernation: bat samples from December (A), January (B), and April (C); roost samples from December (D), January (E), and April (F); far samples from December (G), January (H), and April (I). ASVs are plotted with blue dots if they occur more frequently than predicted by the NCM and orange dots if they occur less frequently. Black solid lines indicate the 95% confidence interval around the predictions. Pie charts depict the distribution of ASVs categorized by neutral processes, more frequently than prediction (“high”), and less frequently than prediction (“low”) during hibernation.

TABLE S1 Fungal infections in bats and environmental samples and group information

Sample id	Average Ct value	*Pd* loads (ng)	Infection status	Time	Site
GeDec1	38.840	9.65e-06	Infected	Dec	bat
GeDec2	NA	NA	Uninfected	Dec	roost
GeDec3	NA	NA	Uninfected	Dec	roost
GeDec4	NA	NA	Uninfected	Dec	bat
GeDec5	NA	NA	Uninfected	Dec	roost
GeDec6	NA	NA	Uninfected	Dec	roost
GeDec7	NA	NA	Uninfected	Dec	bat
GeDec8	NA	NA	Uninfected	Dec	roost
GeDec9	37.830	1.93e-05	Infected	Dec	roost
GeDec10	NA	NA	Uninfected	Dec	bat
GeDec11	NA	NA	Uninfected	Dec	roost
GeDec12	40.670	2.74e-06	Infected	Dec	roost
GeDec13	39.430	6.43e-06	Infected	Dec	bat
GeDec14	NA	NA	Uninfected	Dec	roost
GeDec15	40.465	3.16e-06	Infected	Dec	roost
GeDec16	NA	NA	Uninfected	Dec	bat
GeDec17	NA	NA	Uninfected	Dec	roost
GeDec18	NA	NA	Uninfected	Dec	roost
GeDec19	NA	NA	Uninfected	Dec	bat
GeDec20	45.000	1.40e-07	Infected	Dec	roost
GeDec21	36.800	3.93e-05	Infected	Dec	roost
GeDec22	NA	NA	Uninfected	Dec	bat
GeDec23	NA	NA	Uninfected	Dec	roost

GeDec24	NA	NA	Uninfected	Dec	roost
GeDec25	NA	NA	Uninfected	Dec	bat
GeDec26	NA	NA	Uninfected	Dec	roost
GeDec27	39.020	8.53e-06	Infected	Dec	roost
GeDec28	NA	NA	Uninfected	Dec	bat
GeDec29	NA	NA	Uninfected	Dec	roost
GeDec31	39.970	4.44e-06	Infected	Dec	bat
GeDec33	41.600	1.45e-06	Infected	Dec	roost
GeDec34	NA	NA	Uninfected	Dec	bat
GeDec35	NA	NA	Uninfected	Dec	roost
GeDec36	40.200	3.79e-06	Infected	Dec	roost
GeDec37	NA	NA	Uninfected	Dec	bat
GeDec38	NA	NA	Uninfected	Dec	roost
GeDec39	NA	NA	Uninfected	Dec	roost
GeDec40	NA	NA	Uninfected	Dec	far
GeDec41	NA	NA	Uninfected	Dec	far
GeDec42	41.060	2.10e-06	Infected	Dec	far
GeDec43	39.680	5.42e-06	Infected	Dec	far
GeDec44	40.640	2.80e-06	Infected	Dec	far
GeDec45	NA	NA	Uninfected	Dec	far
GeDec48	NA	NA	Uninfected	Dec	far
GeDec49	NA	NA	Uninfected	Dec	far
GeDec50	NA	NA	Uninfected	Dec	far
GeDec52	NA	NA	Uninfected	Dec	far
GeDec53	NA	NA	Uninfected	Dec	far

GeDec54	39.06	8.30e-06	Infected	Dec	far
GeJan1	NA	NA	Uninfected	Jan	bat
GeJan2	NA	NA	Uninfected	Jan	roost
GeJan3	39.090	8.13e-06	Infected	Jan	roost
GeJan4	NA	NA	Uninfected	Jan	bat
GeJan5	40.100	4.06e-06	Infected	Jan	roost
GeJan6	40.930	2.29e-06	Infected	Jan	roost
GeJan7	NA	NA	Uninfected	Jan	bat
GeJan10	35.550	9.28e-05	Infected	Jan	bat
GeJan11	NA	NA	Uninfected	Jan	roost
GeJan12	NA	NA	Uninfected	Jan	roost
GeJan13	NA	NA	Uninfected	Jan	bat
GeJan14	NA	NA	Uninfected	Jan	roost
GeJan15	40.670	2.74e-06	Infected	Jan	roost
GeJan16	38.190	1.51e-05	Infected	Jan	bat
GeJan18	NA	NA	Uninfected	Jan	roost
GeJan19	NA	NA	Uninfected	Jan	bat
GeJan20	NA	NA	Uninfected	Jan	roost
GeJan21	41.250	1.84e-06	Infected	Jan	roost
GeJan22	39.900	4.66e-06	Infected	Jan	bat
GeJan23	NA	NA	Uninfected	Jan	roost
GeJan24	NA	NA	Uninfected	Jan	roost
GeJan25	38.110	1.60e-05	Infected	Jan	bat
GeJan26	NA	NA	Uninfected	Jan	roost
GeJan27	40.610	2.86e-06	Infected	Jan	roost

GeJan28	NA	NA	Uninfected	Jan	bat
GeJan29	39.050	8.36e-06	Infected	Jan	roost
GeJan30	NA	NA	Uninfected	Jan	roost
GeJan31	40.770	2.56e-06	Infected	Jan	bat
GeJan32	39.550	5.92e-06	Infected	Jan	roost
GeJan33	42.350	8.64e-07	Infected	Jan	roost
GeJan34	38.170	1.53e-05	Infected	Jan	bat
GeJan35	40.080	4.11e-06	Infected	Jan	roost
GeJan36	39.810	4.95e-06	Infected	Jan	roost
GeJan37	NA	NA	Uninfected	Jan	bat
GeJan38	42.040	1.07e-06	Infected	Jan	roost
GeJan39	41.000	2.19e-06	Infected	Jan	roost
GeJan40	38.880	9.39e-06	Infected	Jan	bat
GeJan41	40.145	3.93e-06	Infected	Jan	roost
GeJan42	NA	NA	Uninfected	Jan	roost
GeJan43	NA	NA	Uninfected	Jan	bat
GeJan44	NA	NA	Uninfected	Jan	roost
GeJan45	40.555	2.97e-06	Infected	Jan	roost
GeJan46	NA	NA	Uninfected	Jan	bat
GeJan47	39.160	7.75e-06	Infected	Jan	roost
GeJan49	NA	NA	Uninfected	Jan	bat
GeJan50	40.33	3.46e-06	Infected	Jan	roost
GeJan51	43.395	4.21e-07	Infected	Jan	roost
GeJan52	39.810	4.95e-06	Infected	Jan	far
GeJan53	40.000	4.35e-06	Infected	Jan	far

GeJan54	NA	NA	Uninfected	Jan	far
GeJan55	NA	NA	Uninfected	Jan	far
GeJan56	37.885	1.86e-05	Infected	Jan	far
GeJan57	NA	NA	Uninfected	Jan	far
GeJan58	NA	NA	Uninfected	Jan	far
GeJan59	NA	NA	Uninfected	Jan	far
GeJan61	40.775	2.55e-06	Infected	Jan	far
GeJan62	NA	NA	Uninfected	Jan	far
GeJan63	41.370	1.69e-06	Infected	Jan	far
GeJan64	NA	NA	Uninfected	Jan	far
GeJan65	40.400	3.30e-06	Infected	Jan	far
GeJan66	41.245	1.85e-06	Infected	Jan	far
GeApr1	NA	NA	Uninfected	Apr	bat
GeApr2	NA	NA	Uninfected	Apr	roost
GeApr3	NA	NA	Uninfected	Apr	roost
GeApr4	NA	NA	Uninfected	Apr	bat
GeApr5	NA	NA	Uninfected	Apr	roost
GeApr6	NA	NA	Uninfected	Apr	roost
GeApr7	37.060	3.28e-05	Infected	Apr	bat
GeApr8	NA	NA	Uninfected	Apr	roost
GeApr9	NA	NA	Uninfected	Apr	roost
GeApr10	38.735	1.04e-05	Infected	Apr	bat
GeApr11	37.365	2.66e-05	Infected	Apr	roost
GeApr12	NA	NA	Uninfected	Apr	roost
GeApr13	NA	NA	Uninfected	Apr	bat

GeApr14	40.820	2.47e-06	Infected	Apr	roost
GeApr15	NA	NA	Uninfected	Apr	roost
GeApr16	41.450	1.60e-06	Infected	Apr	bat
GeApr17	39.065	8.27e-06	Infected	Apr	roost
GeApr18	NA	NA	Uninfected	Apr	roost
GeApr19	NA	NA	Uninfected	Apr	far
GeApr20	NA	NA	Uninfected	Apr	far
GeApr22	NA	NA	Uninfected	Apr	far
GeApr23	47.540	2.43e-08	Infected	Apr	far
GeApr24	NA	NA	Uninfected	Apr	bat
GeApr25	NA	NA	Uninfected	Apr	roost
GeApr26	NA	NA	Uninfected	Apr	roost
GeApr27	39.295	7.06e-06	Infected	Apr	far
GeApr28	NA	NA	Uninfected	Apr	far
GeApr29	NA	NA	Uninfected	Apr	far
GeApr30	38.190	1.51e-05	Infected	Apr	far
GeApr31	NA	NA	Uninfected	Apr	far
GeApr32	46.050	1.51e-05	Infected	Apr	far
GeApr33	35.480	6.78e-08	Infected	Apr	far
GeApr34	35.710	8.31e-05	Infected	Apr	far
GeApr35	38.820	9.79e-06	Infected	Apr	far
GeApr36	39.390	6.61e-06	Infected	Apr	far

Note: Sample GeDec52 was removed from subsequent analysis due to low sequence quality.

TABLE S2 Relative abundance of ASVs greater than 0.5% identified in two databases

Feature ID	Phylum	Class	Order	Family	Genus	Final using database	Note
f9c8ca1e9a4f28862a67bfa199653ecf	Ascomycota	Sordariomycetes	Hypocreales	Cordycipitaceae	*Beauveria*	UNITE and Warcup	
762e1475bcc4eb5a2244ba31a2e9f099	Mortierellomycota	Mortierellomycetes	Mortierellales	Mortierellaceae	*Mortierella*	UNITE and Warcup	psychrophilic
020912576a3d3288a9e9094da8322796	Ascomycota	Leotiomycetes	Thelebolales	Pseudeurotiaceae	*Pseudogymnoascus*	UNITE and Warcup	psychrophilic
8a4ba2216d30d3c35494a4e61d1bea1b	Ascomycota	Saccharomycetes	Saccharomycetales	Debaryomycetaceae	*Debaryomyces*	UNITE and Warcup	
53ad2ecac1752f749fd83f1e54a81298	Ascomycota	Sordariomycetes	Hypocreales	Cordycipitaceae	*Engyodontium*	Warcup	
0ccda0b40f247927a9e3d6a156334702	Ascomycota	Leotiomycetes	Thelebolales	Pseudeurotiaceae	*Pseudogymnoascus*	UNITE and Warcup	psychrophilic
1b584a56abb6eb36aa80865d1028e81f	Ascomycota	Sordariomycetes	Hypocreales	Cordycipitaceae	*Beauveria*	UNITE and Warcup	
cfe2f5686b08061bd1b34061e520b847	Ascomycota	Dothideomycetes	Pleosporales	Pleosporaceae	*Alternaria*	UNITE and Warcup	
c745961592ae76bf343e2f0c878d1a06	Ascomycota	Sordariomycetes	Hypocreales	Cordycipitaceae	*Simplicillium*	UNITE and Warcup	
6830678f23580c772dd29fa786d6af79	Ascomycota	Eurotiomycetes	Eurotiales	Aspergillaceae	*Penicillium*	UNITE and Warcup	psychrophilic
415fff2d2a876080a990654d200a8cfd	Mortierellomycota	Mortierellomycetes	Mortierellales	Mortierellaceae	*Mortierella*	UNITE and Warcup	psychrophilic

0f7a845b56465fca87296f8a4969ce39	Ascomycota	Sordariomycetes	Hypocreales	Cordycipitaceae	*Simplicillium*	UNITE and Warcup	
d1f0b7455a0c4386396b4292cd2911cd	Ascomycota	Leotiomycetes	Thelebolales	Pseudeurotiaceae	*Pseudogymnoascus*	UNITE and Warcup	psychrophilic
34f286d144286befe6608efbfe70fb9c	Ascomycota	Sordariomycetes	Hypocreales	Cordycipitaceae	*Simplicillium*	UNITE and Warcup	
64cd532bc879b60acd2f8abe017f7a5e	Ascomycota	Eurotiomycetes	Onygenales	Gymnoascaceae	*Gymnoascus*	UNITE and Warcup	
94c62bb409c336e42216ef0a4a57f252	Ascomycota	Eurotiomycetes	Onygenales	Gymnoascaceae	*Gymnoascus*	UNITE and Warcup	
af42919840537556cd27a86627a62a1e	Mortierellomycota	Mortierellomycetes	Mortierellales	Mortierellaceae	*Mortierella*	UNITE and Warcup	psychrophilic
a1e42fe407d31a6240320a612ea06b0c	Ascomycota	Sordariomycetes	Sordariales	Sordariaceae	*Neurospora*	Warcup	
a7a085c252c329080c47f8a34d864345	Ascomycota	Sordariomycetes	Hypocreales	Cordycipitaceae	*Simplicillium*	UNITE and Warcup	
ee7779796e8d382b4c4595678cc686d3	Mortierellomycota	Mortierellomycetes	Mortierellales	Mortierellaceae	*Mortierella*	UNITE and Warcup	psychrophilic
767705173d6feb71ea26067373c337d3	Ascomycota	Sordariomycetes	Hypocreales	Hypocreaceae	*Hypomyces*	UNITE and Warcup	
38c6ea7faa81514f4b60c446d3b73a84	Ascomycota	Sordariomycetes	Hypocreales	Cordycipitaceae	*Simplicillium*	UNITE and Warcup	
b407b8eebed280b526ca46dac649ed53	Ascomycota	Leotiomycetes	Thelebolales	Pseudeurotiaceae	*Pseudogymnoascus*	UNITE and Warcup	psychrophilic
3f76b35d0d0749bd0cc7c8f16d9a7a01	Rozellomycota						

d0fd5fdac4ba4c752ca29204fbd68afa	Ascomycota	Leotiomycetes	Helotiales	Helotiaceae	*Tetracladium*	Warcup	
10fa32a437d45db26baa7ac7e2a25f33	Ascomycota	Eurotiomycetes	Onygenales	*Onygenaceae*		UNITE and Warcup	
8a114b9b525ae67475e687f010032a1d	Ascomycota	Leotiomycetes	Helotiales	Myxotrichaceae	*Oidiodendron*	UNITE and Warcup	
3a64e8fe2f873f9649ef5f9bbab6abe1	Ascomycota	Sordariomycetes	Hypocreales	Bionectriaceae	*Hydropisphaera*	Warcup	
8833d2b896ed51ec4a8d6dae100d4f3b	Mortierellomycota	Mortierellomycetes	Mortierellales	Mortierellaceae	*Mortierella*	UNITE and Warcup	psychrophilic
ed5d023bedddbf2783271bcf085cd62b	Ascomycota	Leotiomycetes	Thelebolales	Pseudeurotiaceae	*Pseudogymnoascus*	UNITE and Warcup	psychrophilic
f813e9f486347005ac89b3ee13af3354	Ascomycota	Sordariomycetes	Hypocreales	Cordycipitaceae		Warcup	
5f9f5d05cdb0f0f9553b22a5efd4d521	Mucoromycota	Mucoromycetes	Mucorales	Mucoraceae	*Helicostylum*	UNITE and Warcup	
022b155b7ca5cdc67b978e57b1d46863	Basidiomycota	Agaricomycetes	Agaricales	Physalacriaceae	*Flammulina*	UNITE and Warcup	
94120691aaa43f70aabfb864989ccaa6	Mortierellomycota	Mortierellomycetes	Mortierellales	Mortierellaceae	*Mortierella*	UNITE and Warcup	psychrophilic

TABLE S3 W value of differently abundant taxa detected using Analysis of Composition of Microbiomes (ANCOM) comparing different groups

Feature ID	Taxonomy	Time	Site	Infection status

020912576a3d3288a9e9094da8322796	g__Pseudogymnoascus	26	1	NA
022b155b7ca5cdc67b978e57b1d46863	g__Flammulina	33	6	NA
0ccda0b40f247927a9e3d6a156334702	g__Pseudogymnoascus	27	7	NA
0f7a845b56465fca87296f8a4969ce39	g__Simplicillium	33	2	NA
10fa32a437d45db26baa7ac7e2a25f33	f__Onygenaceae	32	1	NA
1b584a56abb6eb36aa80865d1028e81f	g__Beauveria	29	8	NA
34f286d144286befe6608efbfe70fb9c	g__Simplicillium	30	1	NA
38c6ea7faa81514f4b60c446d3b73a84	g__Simplicillium	31	0	NA
3a64e8fe2f873f9649ef5f9bbab6abe1	g__Hydropisphaera	32	2	NA
3f76b35d0d0749bd0cc7c8f16d9a7a01	p__Rozellomycota	28	3	NA
415fff2d2a876080a990654d200a8cfd	g__Mortierella	25	7	NA
53ad2ecac1752f749fd83f1e54a81298	g__Engyodontium	33	1	NA
5f9f5d05cdb0f0f9553b22a5efd4d521	f__Mucoraceae	33	2	NA
64cd532bc879b60acd2f8abe017f7a5e	g__Gymnoascus	33	2	NA

6830678f23580c772dd29fa786d6af79	g__Penicillium	26	5	NA
762e1475bcc4eb5a2244ba31a2e9f099	g__Mortierella	27	5	NA
767705173d6feb71ea26067373c337d3	g__Hypomyces	30	6	NA
8833d2b896ed51ec4a8d6dae100d4f3b	g__Mortierella	30	0	NA
8a114b9b525ae67475e687f010032a1d	g__Oidiodendron	31	1	NA
8a4ba2216d30d3c35494a4e61d1bea1b	g__Debaryomyces	27	33	NA
94120691aaa43f70aabfb864989ccaa6	g__Mortierella	31	12	NA
94c62bb409c336e42216ef0a4a57f252	g__Gymnoascus	32	3	NA
a1e42fe407d31a6240320a612ea06b0c	g__Neurospora	32	1	NA
a7a085c252c329080c47f8a34d864345	g__Simplicillium	30	0	NA
af42919840537556cd27a86627a62a1e	g__Mortierella	31	0	NA
b407b8eebed280b526ca46dac649ed53	g__Pseudogymnoascus	27	2	NA
c745961592ae76bf343e2f0c878d1a06	g__Simplicillium	25	3	NA
cfe2f5686b08061bd1b34061e520b847	g__Alternaria	28	7	NA
d0fd5fdac4ba4c752ca29204fbd68afa	g__Tetracladium	30	7	NA

d1f0b7455a0c4386396b4292cd2911cd	g__Pseudogymnoascus	22	2	NA
ed5d023bedddbf2783271bcf085cd62b	g__Pseudogymnoascus	31	3	NA
ee7779796e8d382b4c4595678cc686d3	g__Mortierella	31	1	NA
f813e9f486347005ac89b3ee13af3354	f__Cordycipitaceae	31	2	NA
f9c8ca1e9a4f28862a67bfa199653ecf	g__Beauveria	26	6	NA
